# Stabilization and Anomalous Hydration of Collagen Fibril under Heating

**DOI:** 10.1371/journal.pone.0078526

**Published:** 2013-11-11

**Authors:** Sasun G. Gevorkian, Armen E. Allahverdyan, David S. Gevorgyan, Aleksandr L. Simonian, Chin-Kun Hu

**Affiliations:** 1 Institute of Physics, Academia Sinica, Taipei, Taiwan; 2 Yerevan Physics Institute, Yerevan, Armenia; 3 Institute of Fine Organic Chemistry, Scientific-Technological Center of Organic and Pharmaceutical Chemistry, Yerevan, Armenia; 4 Materials Research and Education Center, Auburn University, Auburn, Alabama, United States of America; University of Akron, United States of America

## Abstract

**Background:**

Type I collagen is the most common protein among higher vertebrates. It forms the basis of fibrous connective tissues (tendon, chord, skin, bones) and ensures mechanical stability and strength of these tissues. It is known, however, that separate triple-helical collagen macromolecules are unstable at physiological temperatures. We want to understand the mechanism of collagen stability at the intermolecular level. To this end, we study the collagen fibril, an intermediate level in the collagen hierarchy between triple-helical macromolecule and tendon.

**Methodology/Principal Finding:**

When heating a native fibril sample, its Young’s modulus decreases in temperature range 20–58°C due to partial denaturation of triple-helices, but it is approximately constant at 58–75°C, because of stabilization by inter-molecular interactions. The stabilization temperature range 58–75°C has two further important features: here the fibril absorbs water under heating and the internal friction displays a peak. We relate these experimental findings to restructuring of collagen triple-helices in fibril. A theoretical description of the experimental results is provided via a generalization of the standard Zimm-Bragg model for the helix-coil transition. It takes into account intermolecular interactions of collagen triple-helices in fibril and describes water adsorption via the Langmuir mechanism.

**Conclusion/Significance:**

We uncovered an inter-molecular mechanism that stabilizes the fibril made of unstable collagen macromolecules. This mechanism can be relevant for explaining stability of collagen.

## Introduction

**Figure 1:**
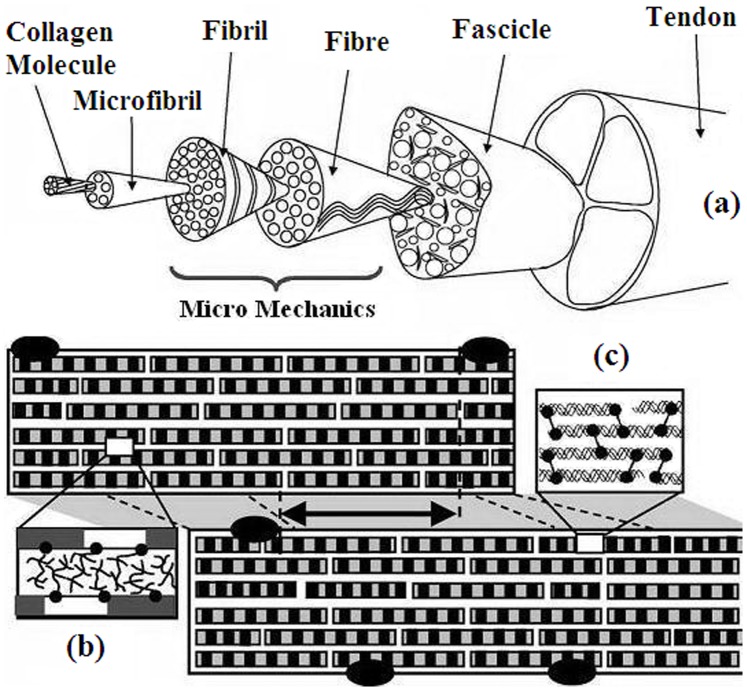
Collagen hierarchy. (**a**) Hierarchic tendon structure [after [43] and [44]]: collagen triple-helices (molecules), micro-fibril, fibril, fiber (fibre), fascicle and tendon. The fascicle is a composite of fibers in a proteoglycan-rich matrix. Fiber is composed of fibrils. Microfibril is a staggered array of collagen triple-helices. The structure of the fibril as consisting of microfibrils is shown in a magnified way. (**b**) The space between microfibril is filled with proteoglycan macromolecules, which form specific bonds between microfibrils. (**c**) Structure of the microfibril as consisting of collagen triple-helices with diameter 1.3 nm and length 300 nm. Covalent bonds between triple-helices are shown in bold.

Type I collagen is the most common protein among higher vertebrates. It forms the basis of fibrous connective tissues, such as tendon, chord, skin, bones, cornea and dentine [1], [2]. Collagen ensures the mechanical stability and strength of these tissues, but its biological role is larger because it also participates in biochemical and immunological protection of the organism [1], [2]. Connective tissues are macroscopic hierarchical structures that consist of several levels: collagen triple-helices (which can be of several types, in particular type I), micro-fibrils, fibrils, fibers and fascicles [1]; see also Fig. 1. Understanding the physical features of these tissues should naturally proceed with disentangling the hierarchy and clarifying the specific roles of each hierarchic level. Though collagen fibers are long-lived stable structures, their constituent collagen triple-helices are marginally (un)stable at physiological temperatures [3]–[6]. For mammals and birds the collagen triple-helix denaturation temperature is adjusted to the body temperature [3]–[6]. This adjustment is made possible by the freedom in the primary structure of the collagen triple-helix. Due to this freedom the collagen is modified to meet the specific needs of tissues as diverse as bones and cornea: each of polyproline left-handed helices (which are super-coiled into the right-handed collagen triple-helix) consists of repeating units of glycine-proline-X or glycine-hydroxyproline-X, where X can be any amino acid (apart from glycine, proline and hydroxyproline) [3]–[6]. The fact of thermal (un)stability at physiological temperatures raises fundamental questions on the relation between collagen stability and levels of its hierarchic structure.

In one way or another such questions were already addressed in literature [4]–[13]. In particular, much attention has been devoted to studying thermal denaturation of collagen fibers via calorimetric methods [4]–[13]. It was argued that while collagen triple-helices are marginally (un)stable at physiological temperatures, the denaturation temperature of collagen fibers and tendons is larger (approximately 60°C at the normal water content) [7]–[13]. This an effect is naturally prescribed to inter-molecular interactions in the higher-order collagen structures, though it is unclear about specific mechanisms: simply entropy reduction effects due to molecular crowding [11], [12], or formation of specific inter-molecular bonds [8]. It was also found that fiber denaturation is a strongly irreversible effect: no indications of native behavior are seen after cooling heat-denatured fiber samples [7]–[13]. Again, while the origin of this irreversibility is loosely prescribed to inter-molecular aggregation, its specific cause is unclear. Note that also the single collagen triple-helix denaturation displays irreversible features, but the causes of this irreversibility have been understood reasonably well [2].

Water is generally expected to be highly relevant for the physics of biopolymers [14]–[17], and collagen is not an exclusion in this respect [3], [18]–[22]. This requires understanding of two different influences: that of the solvent water on the polymer structure and the inverse influence of the polymer on the ambient water [15]. For instance, it is well-known that changing the hydration degree can induce conformational transitions in DNA (A–B transitions) [14], and can even completely block the activity of enzymes [14], [15]. As for the influence of the biopolymer on water, it is known that the polymer surface can change the ordering and dynamical properties of individual, highly perturbed, strongly bound water molecules [15]. It is also known that the structural properties of the water hydrated on biopolymers have several peculiar (glassy) features [15]. Returning to collagen, early calorimetric experiments indicated on unusually high enthalpy of triple-helix denaturation [3], [4], [21], a fact that was related to hydration-bond driven water network around the triple-helix [3], [4], [21]. With time this hypothesis got [22] some further support, but it is still rather controversial [2].

In a somewhat complementary line of research, people studied to which extent specific features of collagen hierarchy are responsible for unique mechanical features of collagen-made tissues [23]–[25]. This subject is relevant for constructing artificial materials having certain biological functions (e.g., artificial heart valves) or materials mimicking certain features of their biological analogues [25].

The present work has three related goals. First, we shall study how the visco-elastic features (Young’s modulus and internal friction) of type I collage fibril change upon heating. It will be shown that visco-elastic features are capable of distinguishing between native and heat-denatured states of the fibril. Moreover, they predict a new intermediate state of the fibril, whose Young’s modulus can become (at sufficiently high temperatures) approximately equal to that of the native fibril. Second, we measured the water content of the native and heat-denatured collagen fibril both as a function of temperature for a fixed pressure, and as a function of pressure for a fixed temperature (hydration isotherms). Besides quantifying the difference between native and heat-denatured fibril, studying hydration with these two settings allowed us to gain understanding of two basic aspects of the water-polymer interactions (how the polymer influences water and *vice versa*). Third, we describe the underlying physics of the above experimental effects by analyzing a theoretical model, which is based on the ideas of the Zimm-Bragg approach to helix-coil transition and the Langmuir approach to the water adsorption.

## Materials and Experimental Methods

The collagen hierarchy is recalled in Fig. 1 [1]. A collagen tendon is made of several fascicles held together. The fascicle consists of fibers, which are made respectively from fibrils, micro-fibrils and collagen triple-helices; see Fig. 1. Each triple-helix consists of three poly-peptide chains wound around each other [1]. As with any hierarchic construction in nature, the criterion of separation into different levels is mainly the interaction strength, e.g., micro-fibrils interact with each other stronger than fibrils do, *etc.*

The axial structure of a microfibril amounts to a staggered array of triple-helices immersed into the proteoglycan matrix [1]; see also Fig. 1. The axial structure is long-range ordered, but it also contains relatively disordered units [1], [27]. Laterally, the fibril is an approximately hexagonal microcrystal; the range of the lateral order is much less than the axial order range [26]. Fibrils and microfibrils are stabilized by several different factors: a small number of covalent links between terminal points of collagen triple-helices, carbonyl-water hydrogen-bonds, hydrophobic and van der Waals interaction between triple-helices, *etc* [1], [2].

Our experimental collagen samples were extracted from Achilles tendons taken from hind legs of young (up to six months old) rats. The extraction was carried out mechanically (without employing any chemical method) in the vivarium of the Yerevan State Medical University (YSMU) located in Yerevan, Armenia. The rats were grown up in the same place. To this end one of the authors (D.S. Gevorgyan) was temporarily employed at YSMU. The whole procedure was approved by the ethical committee of YSMU according to its decision No 12 from 10.11.2009 (as well as by several of its earlier decisions). The members of the ethical committee of YSMU are presented at www.ysmu.am/en/research/574.

All samples were kept in 96% of ethyl alcohol at temperature not higher than 5°C. The logic of our extraction method naturally follows the collagen hierarchical structure shown on Fig. 1. In each extraction stage we employ different micro-tweezers. First, the tendon is broken into pieces by standard medical micro-tweezers right into the ethyl alcohol solution. The diameter of such pieces varies around one hundred micrometers, while their length is around few millimeters. Next we pick up the two ends of the piece by micro-tweezers and carefully shake and pull it. In this way we are able to obtain collagen fibers with diameter around 30 micro-meters. The last stage is separation of the fiber into smaller fragments. This is carried out via self-made micro-tweezers prepared from the wolfram wire (which is also used in electron microscopy). In this stage the fiber is held by wolfram micro-tweezers and separation is achieved by shaking the micro-tweezers in the solution. The whole separation process takes several hours and is monitored by binocular microscope. Note that no longitudinal dissection of samples was employed at any separation stage, so that the method disentangles naturally the collagen hierarchy. In this way several cylindrical samples were extracted with diameters from 1∼14 µm and length 0.3 mm. The (approximate) value of 1 µm for the sample diameter is the smallest one we were able to obtain with the employed method.

It is known that fibril diameter varies widely with temperature, water content and collagen tissue [18], [29]–[31]. The physical or developmental reasons for a large variation among tissues remain poorly understood. It has been suggested that the mechanical properties of tendon are related to the fibril diameter distribution; the large fibrils have a primary role in withstanding high tensile forces and the smaller fibrils have a special ability to resist creep [29]. Fibril diameters also increase with maturation of tissue [30], [31]. The value of the fibril diameter measured via electron microscopy varies from 40 nm till 0.5 µm [1]. For real fibrils this value is underestimated as the electron microscopy demands drying of the studied collagen samples. We studied several samples with the diameter range 1–14 µm and obtained qualitatively similar results. In presenting our results we shall restrict ourselves by the cylindrical sample with the diameter 1 µm and length 0.3 mm. Since this diameter is closer to fibrils than to fibers, we shall call it fibril for definiteness.

The sample under investigation was held by micro-tweezers and washed out in distilled water for twenty-four hours before experiments. It was enclosed in the experimental chamber and placed in a temperature-controlled cabinet with the temperature maintained at 25°C. The hydration level of the sample was adjusted by placing a drop of 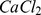 solution at the bottom of the experimental chamber. The sample was allowed to equilibrate at a given humidity for several hours. The relative humidity from 97 to 32% in the chamber was achieved by means of 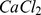 solutions of different concentrations, while the relative humidity of 15 and 10% was obtained via saturated solutions of 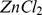 and 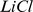, respectively. The chamber was then covered by the heat-insulating jacket and placed on the table of the microscope. The latter is used for measuring sample vibration. The viscoelastic properties and the sample length were measured point by point when varying the temperature continuously at a rate of 0.1°C/min.

The method of measuring the hydrated water content is based on monitoring electrically excited transverse resonance vibrations of wolfram micro-needle with diameter 30–40 µm and length 1.5–2 mm. We pick up the studied fibril sample by the micro-tweezers and reel it on the free end of the wolfram micro-needle [32]. No artificial adhesive is used in this process. The sample is held on the needle by natural adhesive forces, which appear to be so strong that for washing out the micro-needle at the end of the experiment we needed special washing substances.

The method of detecting changes of mass works as follows [32]. Consider resonant transverse vibrations of the console-fixed micro-needle with constant round cross-section. The sample mass 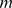 can be obtained via Rayleigh-Ritz formula [33], [34].
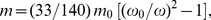
(1)where 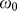 and 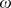 are the resonance frequencies of the needle without and with the sample, respectively, and where 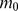 is the mass of the micro-needle. Changing of the sample mass by 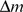 (e.g., due to hydration) leads to changing the resonance frequency from 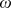 to 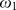. The relative change of mass reads from (1)



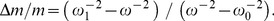
(2)We observe the resonance frequency by changing the frequency of the induced oscillations and looking for the maximal oscillation amplitude of the needle. In experiments we used wolfram micro-needle with platinum sputtering. The micro-needle diameter is 22 µm, and its resonance frequency 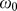 without the sample is around 10 kHz. After fixing the sample on the micro-needle, its resonance frequency changes by 3–5 kHz depending on the sample mass, 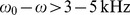. Then we experimental sample is pressurized and subjected to experimental observations. The resonance frequency change due to variation of relative humidity or temperature is within 400–500 Hz. Following to equation (2) let us estimated the error in the relative change of mass 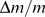 assuming that frequencies are detected with precision 1 Hz. Taking in (2) 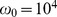 Hz, 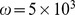 Hz and 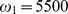 Hz we get that the error in 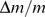 amounts to 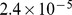.

The micromechanical method for measuring visco-elastic features (the Young’s modulus and the the logarithmic decrement of damping) is also based on observing resonance frequencies. Its details are given in Text S1; see also [26], [28], [35]. The method allows studying samples with diameter ∼1 µm. (This is naturally the lower bound on the diameter within the present method; studying samples with higher diameters is not problematic.) Now the sample (fibril cylinder) is cantilevered by wolfram micro-tweezers from one edge (another edge is free), and its electrically excited transverse resonance vibrations are studied. The free (vibrating) part of the sample is 0.3 mm long (the overall length of the sample is somewhat longer, 0.5 mm).

Recall that the Young’s modulus *E* is defined as the ratio of stress (pressure) over strain. For measuring *E* we smoothly change the frequency f of the induced oscillations and determine the basic resonance frequency, which corresponds to the maximum oscillation amplitude of the sample’s free end. The Young’s modulus of the sample’s main axis reads [56].
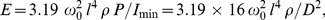
(3)where 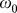 is the resonance frequency, *l* is the sample length, 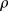 is the density, *P* is the cross-section area, and 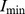 is the main inertia momentum of that section, which corresponds to the deformation plane with minimal stiffness. For the round cross section of our samples 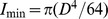 and 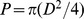, where *D* is the sample diameter. Thus, the Young’s modulus *E* is obtained via (3), where *l*, 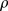 and *D* are known characteristics, and where 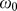 is obtained in experiments.

The logarithmic damping decrement 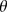 characterizes the strength of internal friction in the sample. It can be defined as
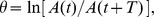
where 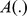 is the decaying oscillation amplitude of the sample after exciting it with the resonance frequency and switching off the excitation, and where 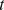 and 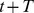 are the times of two consecutive peaks.

**Figure 2:**
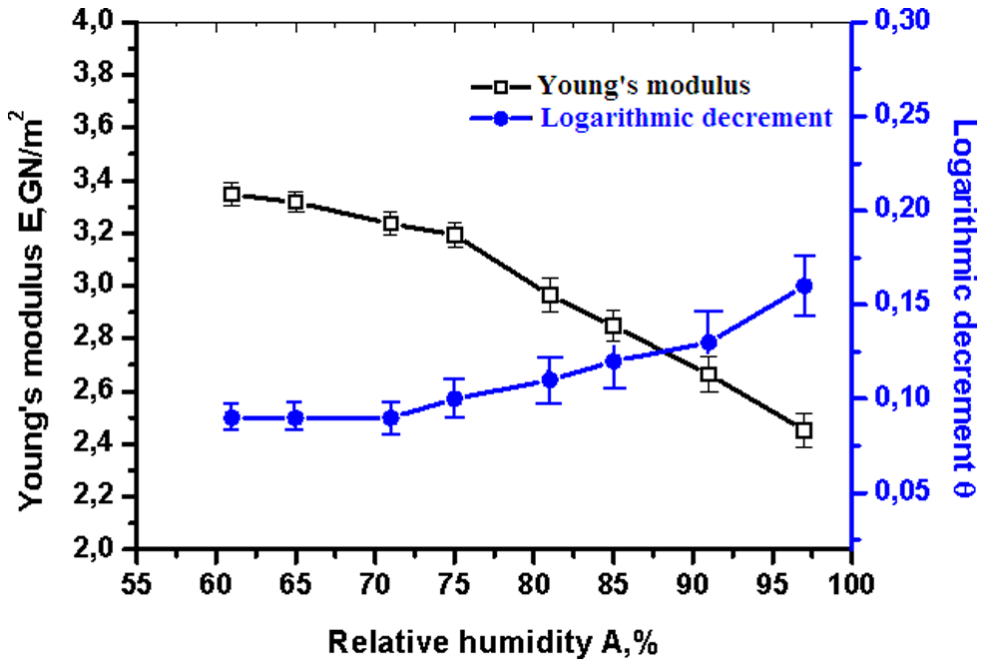
The Young’s modulus and the logarithmic decrement of damping versus relative humidity for a native collagen fibril taken from rat’s tendon. Temperature is fixed at 25 = 97%. We also explicitly display mean-squared errors for each data point.

**Figure 3:**
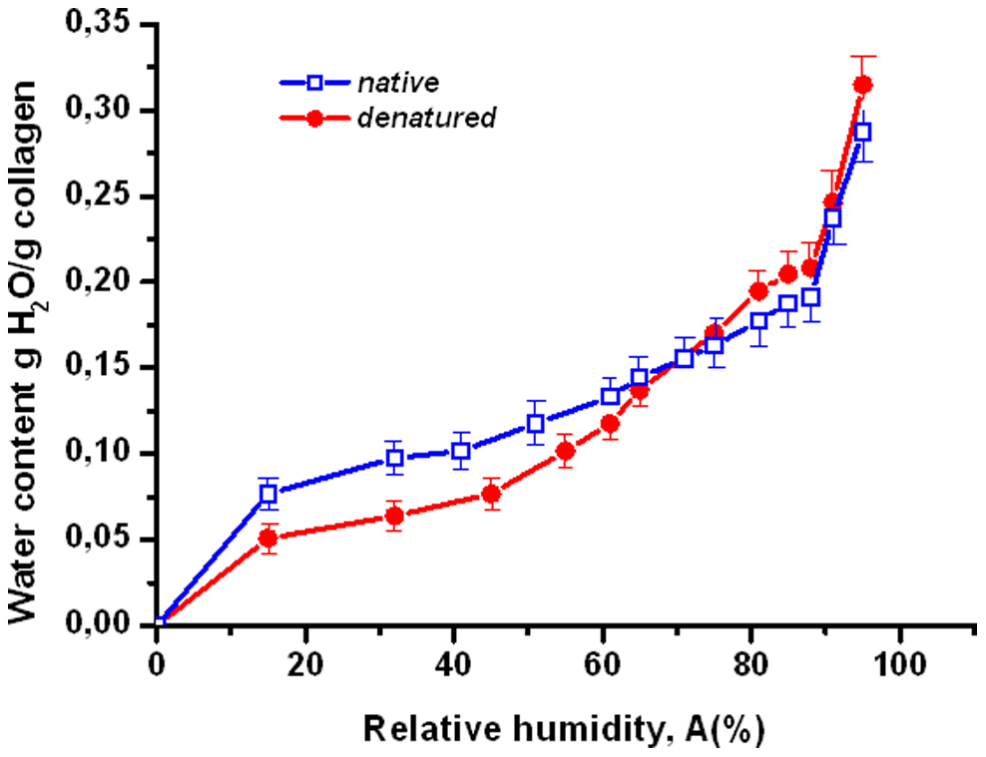
The water content of the native and heat-denatured collagen versus relative humidity (hydration isotherms) for collagen fibril taken from rat’s tendon. Temperature is fixed at 25°C. Experiments were carried out starting from the highest relative humidity A = 97%. We also explicitly display mean-squared errors for each data point.

**Figure 4:**
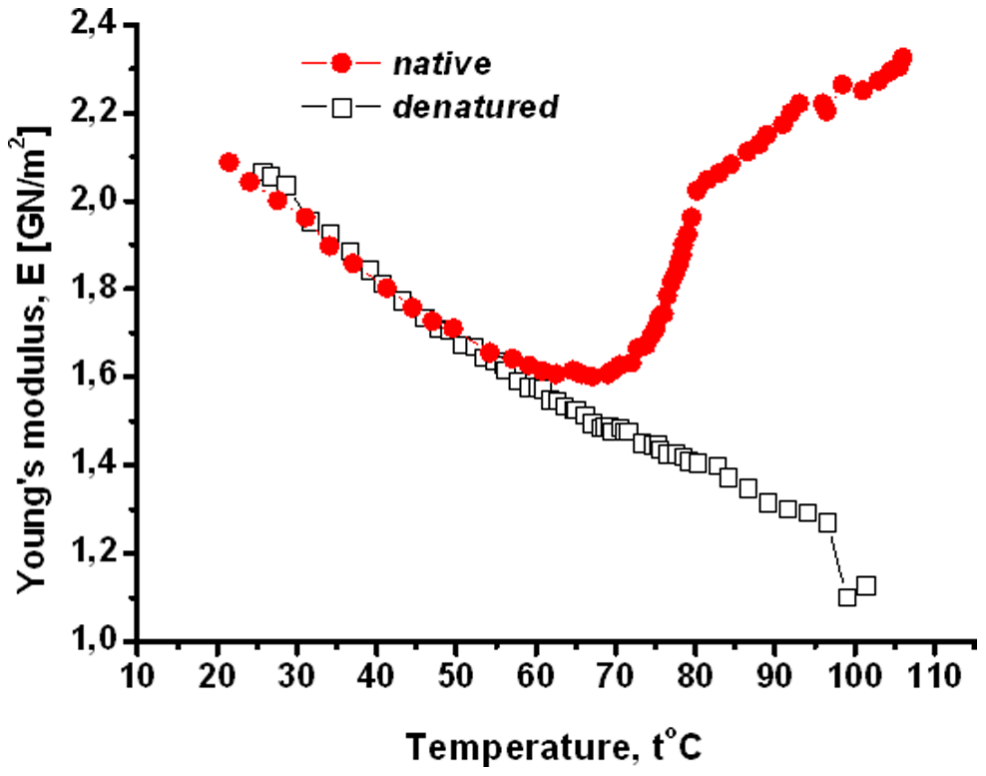
The Young’s modulus versus temperature for the native and heat-denatured collagen fibrils. In all experiments the heating speed is 0.1°C/min. In all experiments the heating speed is 0.1°C/min.

Equation (3) implies several sources of error for determination of the Young’s modulus *E*. First, let us estimate the error coming from measuring the resonance frequency 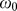. Our method normally works on frequencies up to 10 kHz. Typical frequency changes (e.g. due to hydration) are up to few hundred Hz. Assuming for clarity that the accuracy of the frequency observation is 1 Hz (the actual accuracy is certainly larger) we get that error for calculation of the Young’s modulus is around 0.1%. Our experimental data for collagen fibril is presented in Figs. 2–4. In Fig. 4 this error can be considered to be included in the data point size. Next source of errors comes from evaluation of the sample diameter, which we measure with the accuracy of 0.02 µm. This precision was achieved via Linnik interferometer (MII-4, LOMO); at several points we controlled the results via Research Inverted System Microscope OLYMPUS IX71 with DIC, which has phase contrast on image expansions 20x and 40x. For a sample with diameter 1 µm the error in *E* is about 2–3%. The relative error coming from evaluation of the sample length *l* is less significant, because for our samples *l>D*. We note however that to 2–3% error refers to the absolute value of the Young’s modulus. For the relative change 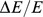 of the Young’s modulus on the same sample the error is expected to be smaller, since we did not note any systematic change of the sample diameter and length with hydration and temperature variation.

## Experimental Results and Discussion

### Influence of Humidity Changes on the Fibril Structure

Fig. 2 reports the Young’s modulus and the logarithmic decrement of damping versus relative humidity for the collagen fibril. The temperature was held constant at 25°C. Expectedly, under increasing the relative humidity, the fibril becomes less rigid (the Young’s modulus decreases) and more viscous (the logarithmic decrement increases). It is known that for other biopolymers – e.g., globular proteins, amino acid crystals and DNA – the Young’s modulus can change over several orders of magnitude as a function of humidity [14]. Collagen fibril is seen to be different in this aspect, because the dependence of the Young’s modulus and the logarithmic damping decrement on the water content is not strong; see Fig. 2. Moreover, this dependence did not qualitatively change after irreversible heat-denaturation of the fibril (the heat-denaturation was carried out holding the fibril at 120° for several hours; see below for more details).

These results are hinting that for considered temperatures and humidity the influence of hydration on the fibril structure is going to be less important than the influence of the fibril on the hydrated water. Below we shall confirm this hint via theoretical approach.

### Hydration Isotherms

Fig. 3 displays hydration isotherms of native and denatured collagen fibril at 25°C. They resemble the isotherms of type III under the classification of Brunauer, Emmett and Teller (BET) [32], [33]. This type of isotherms are frequently encountered for biopolymers in solid phase [36], [37]. For humidity between 20% and 60% the water content (almost) saturates, because all adsorption centers on the polymer are occupied. Thus the first adsorption layer is formed (this corresponds to well-bound water). For even a larger humidity second and higher adsorption layers are displayed; this happens primarily due to water-water interaction [36].

### Dependence of the Young’s Modulus on Temperature

Fig. 4 describes the behavior of the Young’s modulus as a function of temperature. We separated five characteristic intervals for this quantity.

**Figure 5:**
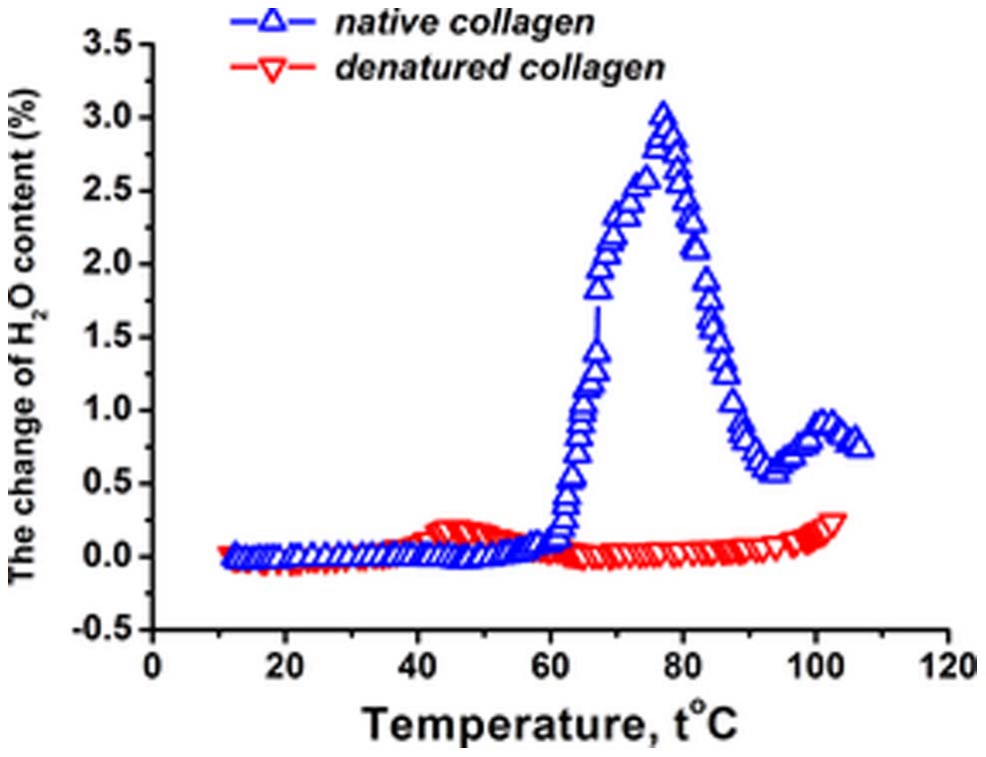
The hydrated water content versus temperature for the native and heat-denatured collagen fibril. The initial temperature and water content in the chamber 25°C and 93%, respectively. At this temperature and humidity the water content of the native and denatured fibril is h = 0.3 g water/g of dry collagen and h = 0.22 g water/g of dry collagen, respectively. The change of the water content is given in percents relative to that water content at 25°C. The heating speed is 0.1°C/min.

1. The Young’s modulus of the native collagen smoothly decreases between 20° and 45°C; see Fig. 4. There is no difference between the Young’s modulus of the native sample and that of the heat-denatured sample. The conformational changes in this interval are reversible, since the features of the fibril did not change after repeating the cooling-reheating process ten times. It is likely that in this stage only the triple-helical conformation changes.
2. In the temperature interval 45–58°C the decrease of the Young’s modulus for the native sample is impeded as compared to the previous stage; see Fig. 5. The Young’s modulus of the native sample is larger and decreases slower as compared to that of the heat-denatured sample. So far the behavior of the Young’s modulus was intuitively expected (decreasing under heating).

Also in this interval we noted first indications of hysteresis and irreversibility during heating and re-cooling (not shown on figures). Similar aspects were explored in Ref. [57], where it was shown that the native collagen fibril has certain features of the glassy state.

1. The third interval lies in 58–75°C. Here the Young’s modulus of the native fibril is nearly constant. To our knowledge such an effect was never seen for a single collagen triple-helix (or for a dilute solution of triple-helices). In particular, the helix-coil transition of the (isolated) type I collagen triple-helix is known to proceed without intermediates [49]. Thus the approximate constancy of the Young’s modulus in this temperature interval should be due to activation of intermolecular interaction: partially restructured collagen molecules start to overlap and create new contacts between each other. This hypothesis is consistent with experimental results on the mutual interaction of two collagen triple-helices in water, which also demonstrated that the intermolecular interaction is activated at intermediate temperatures [38]. For the experiments carried out in [38] these temperatures were around 35°C, but for the present situation, where the collagen triple-helices are arranged into fibrils, their entropy is reduced, we expect such activation temperatures to be higher. The approximate constancy of the Young’s modulus for considered temperatures means that the rigidity decrease due to the structure denaturation is compensated by the rigidity increase due to formation of inter-molecular bonds.

In the considered temperature interval 58–75°C the hysteresis is more pronounced than for the previous one. Note that the Young’s modulus of the heat-denatured sample continues to decrease following the same linear rule as for the previous stages; see Fig. 5. Thus, the heat-denatured sample does not feel the temperature interval 3.

1. In the temperature interval 75–80°C the Young’s modulus of the native sample starts to increase with temperature in sharp contrast with the Young’s modulus of the heat-denatured sample that keeps decreasing. At the end of this interval (i.e., at 80°C) the Young’s modulus almost approaches its initial value at 25°C; see Fig. 4. A possible reason for the increase of the Young’s modulus is that the network of the inter-molecular contacts develops and contributes significantly to the rigidity.

In this context we note that the coincidence of the Young’s modulus for the native and heat-denatured sample at the *physiological* temperatures 20–45°C (temperature interval **1**) is a rather non-trivial fact. It may be related to the functioning of a burned and scared tissue, which for physiological temperatures is supposed to have at least some features of the native tissue.

1. Above 80°C the Young’s modulus keeps on increasing, though slower than for the previous step. The dynamics in this region is fully irreversible: if the heating is stopped at some temperature higher than 80°C, the fibril slowly (within several days) relaxes to the heat-denatured state. During this relaxation the Young’s modulus has to decrease; see Fig. 4. We monitored the length and diameter of the sample during the whole process (including heating from 20°C), and did not detect any essential change in both quantities.

Thus we see that upon heating the collagen fibril enters into a meta-stable state, which is intermediate, i.e., neither native, nor properly heat-denatured. We shall see below that this state has non-trivial features with respect to water adsorption.

### Changes of Water Content Under Heating

**Figure 6:**
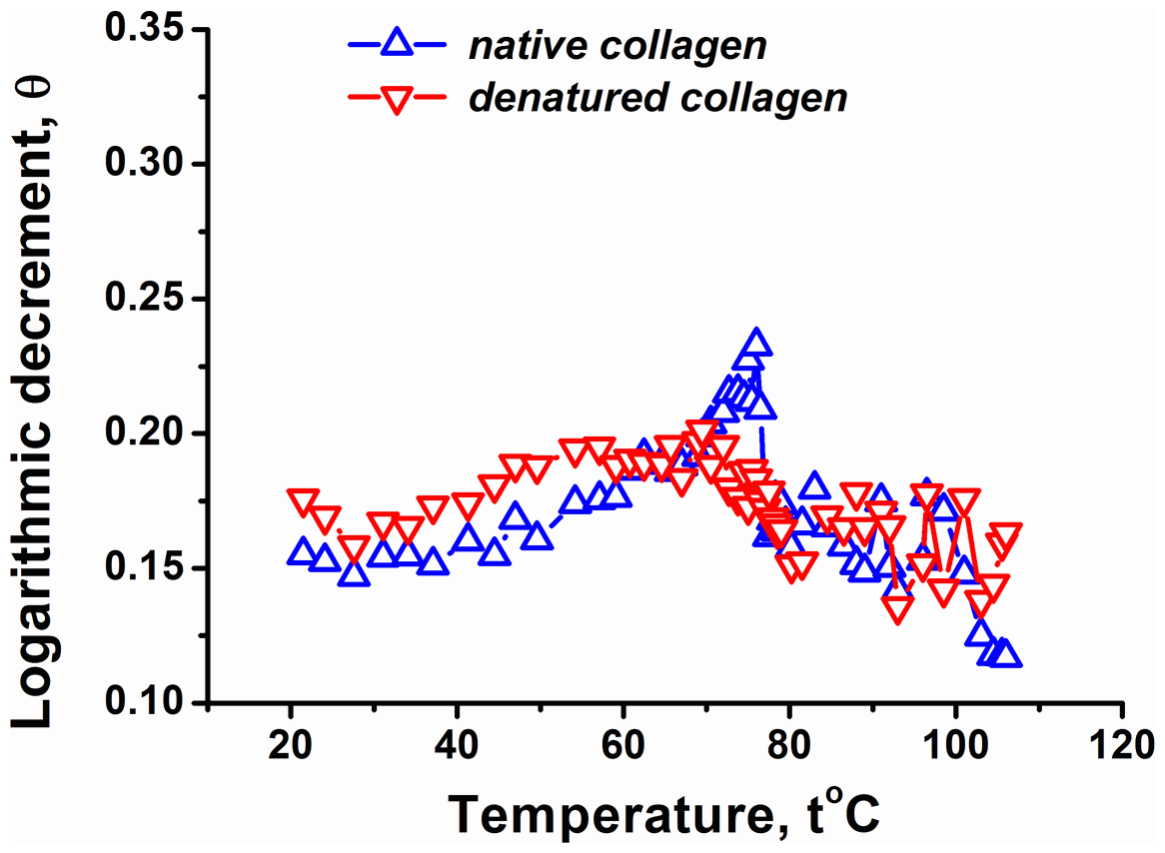
The logarithmic damping decrement versus temperature for the native and heat-denatured collagen fibril. The initial temperature and water content in the chamber is 25%, respectively. The heating speed is 0.1 C/min.

Fig. 5 shows the water content change of collagen fibril under heating (the pressure is held constant). The water content is nearly constant for temperatures lower than 58°C. This behavior is not specific to the native fibril, and is reproduced for the heat-denatured situation. Recall that for temperatures lower than 58°C the Young’s modulus reversibly decreases upon heating. Obviously, the adsorbed water follows reversible changes of the fibril without opening new adsorption centers. This is also seen on the logarithmic decrement of damping, which for temperatures lower than 58°C displays more or less constant behavior; see Fig. 6.

The surprising point comes in the temperature interval 58–75°C. Recall that in this regime the Young’s modulus is nearly constant and it increases for higher temperatures; see Fig. 4. While it is expected that the water content will monotonously decrease during heating (due to evaporation), Fig. 5 shows a completely different pattern: the hydrated water content of the native collagen fibril sharply increases in the temperature interval 58–75°C. The maximum of the hydrated water content is around 75°C. This absorption of an additional amount of water instead of evaporating it in the heating mode can be due to internal restructurization of the fibril. This is the same structural effect that ensured the approximately constant Young’s modulus in the regime **3**; see Fig. 4 and our discussion above. Note that the presence of structural changes is also indicated by the behavior of the logarithmic decrement of damping, which shows a pick in the temperature interval 58–75°C; see Fig. 6.

The additionally absorbed water should be connected to the adsorption on the first hydration layer, where the molecule-water interaction is the strongest one. It is likely that the hydrated water content increases, because new adsorption centers open up during partial restructuring of triple-helices. The non-monotonous changes of the hydrated water content and of the Young’s modulus are absent for the denatured fibril; see Figs. 6. Thus, the native character of the fibril is crucial for the water accumulation effect.

Note that for temperatures higher than 75°C the water content decreases, while the Young’s modulus starts to increase; see Fig. 4 and Fig. 5. The theoretical analysis presented in section 6 will identify evaporation as the primary source of the water decrease. These two effects are related, as witnessed by the fact that in the vicinity of 85°C the water content again changes non-monotonously, and this is reflected in the behavior of the Young’s modulus, whose increase is impeded; see Fig. 4 and Fig. 5.

Such a relation between the water content and the Young’s modulus can have two reasons. On one hand, the water can act as a lubricant with its evaporation leading to the rigidity increase. On the other hand, thermal degradation of the fibril structure may facilitate the hydrated water content decrease due to evaporation. The difference between these two mechanisms is that in the first case the water is supposed to drive the conformational changes, while in the second scenario the conformation is the driver. At the level of experimental results it is difficult to tell which mechanism is really at play, though our discussion on absorption isotherms indicated that the influence of the water on the fibril structure is not essential. The theoretical scheme presented below will be seen to support the second mechanism (conformation influences the adsorbed water).

### Discussion of Experimental Results

We shall now succinctly discuss the obtained experimental results and put them in the context of existing literature.

1. As we already mentioned in the introduction, there is clear calorimetric evidence on the structural changes of collagen fibers and tendons taking place around 60°C [6]–[13] (this temperature depends on the heating rate; presumably for this reason Ref. [7] reporting on the adiabatic calorimetry with considerably smaller heating rates locates the characteristic temperature around 50°C). This is associated with denaturation temperature of fibers, and it is clearly higher than the temperature 37–40°C, where an isolated triple-helix denaturates [2], [3], [4]. Some calorimetric experiments on specially prepared collagen solutions show simultaneously two different peaks of specific heat at temperatures 40° and 60°C [3], [10]. At the temperature 59°C our results on the Young’s modulus also indicate on the beginning of structural changes, whose interpretation is roughly consistent with early stages of the denaturation process: triple-helices start to unfold and activate inter-molecular bonding interaction due to which the Young’s modulus stabilizes. This picture is also consistent with the water absorption due to newly opened centers. However, our results also indicate that the native fibril does not immediately turn into the denatured state. There is a meta-stable intermediate state, whose Young’s modulus increases with temperature. This intermediate state decays into the denatured state very slowly and at higher temperatures.

We should stress that the denaturation transition of collagen fibril differs from analogous transitions found in globular proteins and DNA, because in those cases the Young’s modulus sizably and suddenly decreases due to formation of random coil out of the ordered structure; see, e.g., [39]. Due to the random coil formation also the size of experimental samples increases significantly. These effects are not seen on collagen fibril.

1. Esipova and coauthors conjectured that the collagen triple-helices dehydrate during formation of collagen (micro)fibrils [19]. This conjecture was supported by rather indirect experimental observations [19]. Our result on the water absorption in the temperature interval 58–75°C is consistent with this conjecture, because if the triple-helices dehydrate during the fibril formation, they should re-hydrate during partial denaturation of the fibril.
2. Mreshvili and Sharimanov studied calorimetrically the water content change of triple-helical collagen during denaturation [21]. They observed that the overall water content increases after complete denaturation. On the other hand, employing NMR experimental results they conclude that the concentration of the strongly bound water decreases after complete denaturation. The decrease of the strongly bound water after denaturation is consistent with our observations; see Fig. 3, where the first hydration layer of the heat-denatured fibril is smaller than that of the native sample. The overall increase of the water content after denaturation is not consistent with our observations.
3. Bull reported on the adsorption isotherm for collagen at temperature 25°C [15], [40]. For relative humidity lower than 30–40%, these results agree with our finding for the absorption isotherm of collagen fibril; see Fig. 3. For higher humidity we predict lower water contents. However, the main qualitative difference is that for intermediate humidity (between 20 and 80%) the isotherm obtained by Bull predicts a linear behavior of the water content and does not display a visible saturation; compare with Fig. 3 which does show a saturation effect for intermediate humidity. One reason for this difference is that we focused on collagen fibril, while Bull studied collagen tendons, which have more sites (in between of fibrils) for water uptake.
4. Pineri and coauthors studied hydration of collagen tendons via calorimetric and mechanic experiments for temperatures lower than 300°K [41]. This overlaps with our temperature regime only in a narrow interval of 5–10°C. However, even within this narrow interval the results by Pineri and coauthors on the relative rigidity are consistent with our observations on the Young’s modulus versus temperature: the modulus decreases with temperature and water acts as a plasticizer; see Fig. 4.
5. Mesropyan and coauthors studied dehydration of collagen tendon within temperature range 20–100°C via calorimetric methods [42]. They did not see any non-monotonous change of the water content (see our Fig. 5), presumably because their measurement points are rather sparse (e.g., there are no water content data between 45° and 75°).

### Theoretical Results and Discussion

The purpose of the following theoretical consideration is to come up with the simplest equilibrium model of the fibril, which under natural assumptions of polymer physics (helix-coil transition, Zimm-Bragg approach [45], [46], [47], Langmuir adsorption [37]) will model the dominance of intermolecular interactions under increasing the temperature, and as a result of this dominance will lead to non-monotonous water adsorption. We shall not attempt at quantitative data fitting, since the approach though supposedly the simplest one still contains several free parameters with unknown experimental values. Instead, we shall point out at the naturalness of the model. Below we shall compare this approach with other models of inter-molecular interactions known in literature. At any rate, this approach is based on equilibrium statistical mechanics and it is not supposed to describe irreversible aspects of heat-denaturation. Another drawback of the approach is that it does not account directly for elastic modules.

A theoretical model presented below describes the fibril as a collection of collagen triple-helical macro-molecules. Each molecule is treated via the usual logics of the Zimm-Bragg approach, i.e., it is represented as a chain of units that can be in several conformational states [45], [46], [47]. Within the standard Zimm-Bragg model of the helix-coil transition, each unit can be in helical or in coiled state [45], [46], [47]. Though restricting to just two conformation states is certainly an idealization, it sufficed for the main purpose of the Zimm-Bragg model, i.e., to provide a simple phenomenological description of the helix-coil transition in biopolymers [45], [46], [47]. The Zimm-Bragg model was already applied for describing the helix-coil transition in isolated type I collagen triple-helices, and gave an adequate explanation of experimental results [48], [49].

For the present situation it is reasonable to introduce yet another state for the conformational unit of the separate triple-helix, where the unit is not properly helical, but instead it participates, e.g., via hydrogen-bonds, in building up the inter-molecular structure of the fibril. We saw above experimental evidences for such a third state between helix and coil. Thus, each macromolecular unit can be in three conformational states, which we denote 1 (perfect helix), 0 (intermediate state participating in inter-molecular structure) and −1 (coil).

Restricting ourselves to binary intermolecular interactions, the free energy of the fibril reads
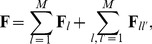
(4)where 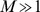 is the total number of molecules in the fibril, and where 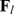 and 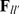 refer, respectively, to the free energy of a single molecule and binary (two-molecule) interactions. We employ the Ising representation of the Zimm-Bragg model [45] and propose the following expression for the single-molecule free energy
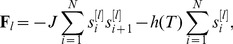
(5)where 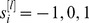 refer to the conformational state of a single unit in the given molecule 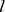, 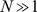 is the total number of units for each molecule, 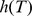 accounts for the free energy of the unit, while 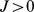 describes cooperativity (i.e., unit-unit interaction). Within the general spirit of the Zimm-Bragg approach [45], [46], [47] we assumed that each helical (resp. coiled) unit agitates its neighbor units on the same molecule to be also helical (resp. coiled). This implies that 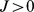. However, no copperativity is assumed for the intermediate state: if 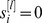, then no gain or loss of free energy is assumed by the unit-unit interaction for any value of 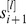. This is natural, because the intermediate state is supposed to be driven and supported mainly by inter-molecular interactions, whereas the cooperativity is supported by a single-molecule backbone.

As for the single-unit free energy 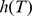, we shall make the same experimentally supported assumption as in the standard Zimm-Bragg model, i.e., we assume that 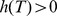 below the helix-coil transition 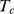, and 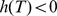 for 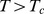 [45], [46], [47]. In the vicinity of 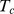, the behavior of 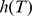 is naturally linear [45], [46], [47]:
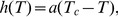
(6)where 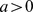 is a dimensionless constant of order 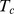 (we assume 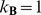). In other words, the single-molecule contribution 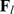 supports helix (coil) for 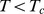 (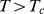).

Let us now turn to intermolecular free energy 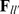, which combines interactions of different origin: weak electrostatic, hydrophobic, van der Waals and covalent linking (the latter operates mainly at the end-points of the two triple-helices). It is known that the interaction between two collagen molecules is strongly repulsive at short distances, and long-range attractive at moderate and long distances [38]. Thus,
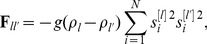
(7)where we assumed that the strength 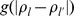 of the inter-molecular interaction depends only on the absolute value of the cylindrical radius-vectors difference 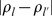 of two molecules. The assumption on the cylindrical symmetry is natural, because the collagen triple-helices are strongly stretched within the fibril. Note that a positive 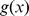 means that the free energy (7) suppresses the intermediate state 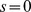 in favor of completely helical or completely coiled states 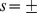
1. In contrast, a negative 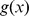 supports the intermediate state. Thus, according to the above discussion, 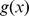 is strongly positive for 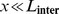, where 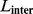 is the mean inter-molecular distance in the fibril, and 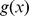 is negative (with a long-range tail) for 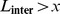. The factor 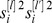 in (7) naturally indicates that the inter-molecular interaction drives the intermediate state for each unit.

For 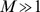 (a large number of triple-helices in the fibril) we can employ the long-range nature of 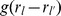 and apply the mean-field approach with respect to inter-molecular interaction; see [50] for a general introduction to the mean-field method. It is well-known that this approach produces external field acting on a single (effective) molecule [50]. Thus for the effective free energy we get
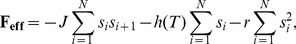
(8)where the parameter 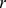 generally depends on temperature and should be determined self-consistently from the minimization of 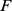 in (8) given the explicit form of 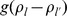 (which by itself is temperature-dependent [38]). One can show that for our purposes the dependence of 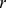 on the temperature is relatively weak. Thus, we assume that for the relevant range of parameters 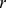 is a negative constant; see the above discussion on the sign of 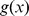.

Now it is time to discuss how the model interacts with water. The free energy 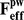 of the polymer and the adsorbed water on the first hydration layer reads from (8)
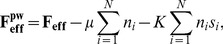
(9)where 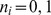 indicates on the presence or absence of water molecule on the macromolecular unit 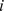, 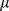 is the chemical potential of the adsorbed water (this terms corresponds to the standard Langmuir adsorption theory [37]), and where 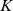 describes the water-conformation interaction. The form of this interaction for 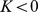 assumes that at the intermediate state (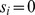) new adsorption centers are available, while the complete melting (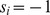) creates even more such centers. Conversely, the presence of water tends to destabilize the helical state towards the intermediate state. In writing down (9) we neglected the *direct* interaction between adsorbed water molecules, since for the considered situation we expect it to be much smaller than the water-conformation interaction. We also assumed that each unit can support only one molecule of water. This assumption is somewhat unrealistic, but we adopt it for simplicity, because no qualitative changes were detected upon increasing the number of water molecules on each unit. For collagen triple-helices this number varies between1 and 10 [1], also depending on how the conformational unit is defined (35% hydration means for a triple-helix that there are approximately 5 water molecules per amino-acid).

In the statistical sum
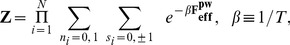
(10)the summation over 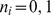 can be taken directly. This leads to the free energy 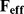 in (8) with substitutions



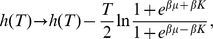


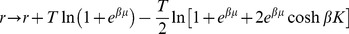



Now the statistical sum 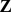 is treated in the thermodynamic limit 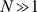 via the standard transfer-matrix method [45], [46], [47]. The equilibrium averages 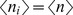, 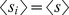 and 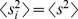 are calculated by differentiating 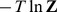 over, respectively, 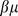, 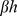 and 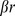. The concentration 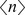 of the adsorbed water can be conveniently expressed via 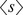 and 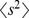:
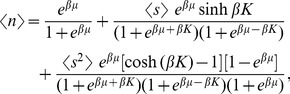
(11)where the factor 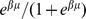 in 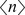 is what will come out in a free (i.e., without water-polymer interaction) Langmuir theory of adsorption [37], and where 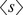 and 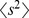 are found from solving a cubic equation, which is generated by the transfer-matrix method. (For the ordinary Zimm-Bragg model one generates in a similar way a quadratic equation [45], [46], [47].) We do not write down this cubic equation and its solutions. The order parameters of this problem are the probabilities (or fractions) of various states:
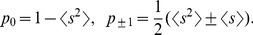
(12)


### Discussion of Theoretical Results

**Figure 7:**
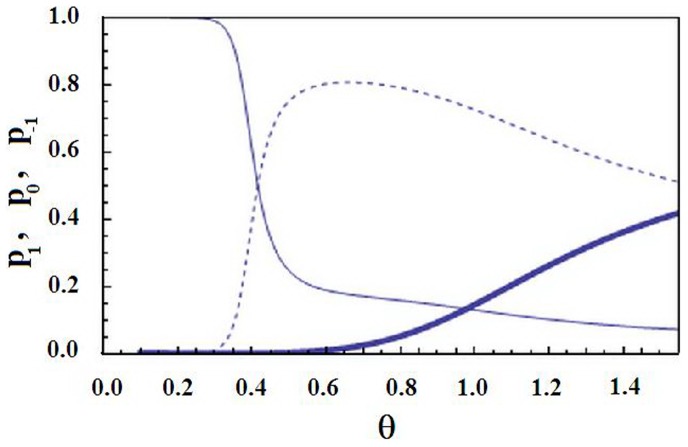
The order parameters of the generalized Zimm-Bragg model; see (12). 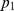
(normal line), 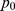 (dotted line) and 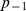 (thick line) versus the dimensionless temperature 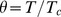 for 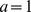, 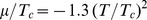, 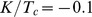, 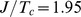, 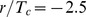.

Note from (9–11) that all the parameters (including temperature) can be put in a dimensionless form 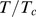, 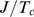, 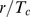 (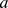 is already dimensionless) and that 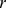 in (8) is negative. Since we are going to study the heating regime, we assume that the chemical potential 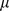 of the adsorbed water is negative, and that 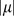 increases with temperature 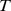 faster that 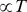. Then the pure Langmuir contribution 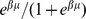 decreases with increasing temperature [37], as it should be for the evaporation. The simplest assumption we adopt is 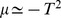 for the pertinent range of temperatures. Fig. 7 displays the behaviour of order parameters (12). The parameters are chosen such that there is visible cooperativity along the chain (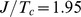), the intermolecular interaction is sufficiently large (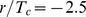), while the conformation-water interaction is weak 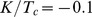. It is seen that for low temperatures the state1 dominates (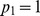 ), i.e., all units are helical. Around 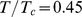 there is a relatively sharp transition from the helical state to the intermediate state: upon decreasing the temperature 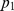 suddenly changes from 1 to 0.2, while 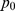 raises from 0 to 0.8. The fraction of coiled states is negligible around this transition (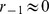); see Fig. 7. This transition is naturally driven by the intermolecular interaction 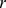, since it is absent for smaller values of 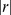. For larger temperatures the fraction of coiled units 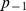 grows gradually (i.e., without sudden transitions), while 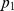 and 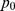 gradually decay; see Fig. 7.

For smaller values of 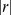 we revert to the standard helix-coil transition scenario *a la* Zimm-Bragg [45], [46], [47]: at 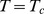, 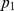 suddenly jumps from 1 to a small value, 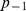 suddenly jumps from zero to almost 1, while the intermediate state 0 is never significantly populated: 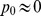. It should be clear that the sudden character of this transition (for small or large values of 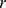) is related to the cooperativity parameter 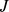; transitions are smeared for a smaller 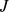.

**Figure 8:**
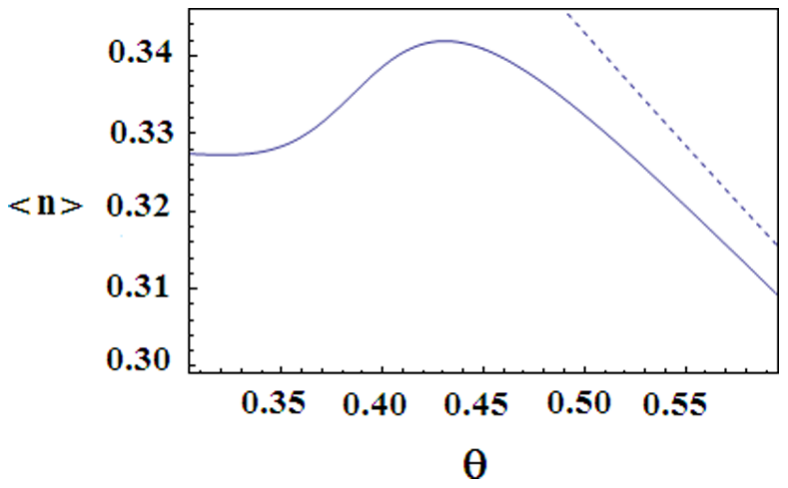
The adsorbed water content of the Zimm-Bragg model. Normal line: the average number 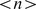 of adsorbed water versus the dimensionless temperature 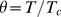 for the same parameters as in Fig. 9. Dashed line: 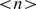 versus 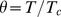 for the same parameters as in Fig. 9, but with 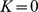 (no interaction between the water adsorption and the conformation).

Thus we see that a sufficiently strong intermolecular interaction can lead to dominance of the state 0 at intermediate (not high and not low) temperatures. Now at this state the water adsorption is facilitated (as compared to the fully helical state) and although without coupling to the conformation the water content will monotonously decay (see Fig. 8), even a sufficiently weak water-conformation coupling will lead to a local maximum in the behavior of the adsorbed water; see Fig. 7 and Fig. 8. The water content maximizes in the same narrow range of temperature as the transition from the helical state 1 to the intermediate state0; compare Fig. 7 with Fig. 8. For lower temperatures the adsorbed water concentration 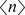 is nearly constant (in agreement with experiments; see Fig. 6), while for higher temperatures it monotonously decays, simply because temperature becomes too high and evaporation becomes the dominant process. Recall that the chemical potential behaves as 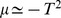 facilitating the evaporation. Thus we qualitatively reproduced the non-monotonous behavior of the adsorbed water content seen in our experiments.

Let us put the water-conformation interaction 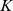 to zero. (Recall that *K* was already assumed to be small during the above discussion.) Then the behavior of order parameters 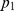, 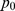 and 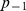 will not change much, only the temperature of the sudden transition from the state 1 to the state 0 will slightly decrease (not shown on figures). In contrast, the behavior of the adsorbed water content changes crucially, because for 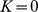 the density 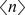 of adsorbed water will monotonously decay upon increasing the temperature; see Fig. 8. Thus conformations influence the adsorbed water, while the inverse influence is small.

### Relations with Previous Works

1. Miles and Ghelashvili employed the polymer-in-box mechanism for explaining the stability of collagen fibers versus relative instability of a separate triple-helix [11]. They imagined that inter-molecular interactions amount to creating an effective cage for each separate triple-helix. This gives adequate predictions for collagen fiber stability in solvent [12]. However, the polymer-in-box mechanism misses an important ingredient: the inter-molecular interactions inside of the fibril are activated at a specific temperature range, and this activation leads to restructurization of the fibril.
2. Maleev and Gasan considered the effect of inter-molecular interaction on the helix-coil transition in dilute and condensed solutions of polypeptides [51]. They argued that partially molten helical segments belonging to different macromolecules can form additional hydrogen bonds and thus lead to formation of irregular 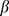 sheet structures. It was shown that inter-molecular interactions generally reduce the cooperativity of the helix-coil transition [51]. This aspect agrees with our results.
3. Sandler and Wyler applied the Zimm-Bragg model to thermal helix-coil transition in collagen fibers [52]. They endow each triple-helix with (mean-field) orientational degrees of freedom, and come up with a phenomenological free-energy for the inter-molecular interaction, which accounts only for the excluded-volume repulsion between the triple-helices [52]. Within such an approach it is not possible to distinguish between the usual helix-coil transition (where each separate triple-helix transits from the helix to coil) and the state, where intra-molecular segments of an intermediate helicity stabilize each other due to inter-molecular interactions, a basic point of our theoretical approach.
4. There are several models in literature devoted to helical bundles, i.e., few (one or two) interacting helical macromolecules such as paramyosin and tropomyosin [53], [54], [55]. These models make various generalizations of the Zimm-Bragg approach in accounting for the inter-molecular interaction. It is shown that certain features of this interaction may result in cooperativity increase, or even lead to new scenarios of helix-coil transitions [55].

## Summary

In this paper we studied the Young’s modulus, the hydrated water content and the logarithmic decrement of damping for collagen fibrils. We wanted to understand the specific mechanisms that govern the stability of collagen fibrils under heating. The relevance of this problem stems from the fact that separate triple-helical constituents of the fibril are unstable at physiological temperatures [2]–[5], so that the notorious stability of collagen-based structures should be based on specific inter-molecular interactions.

The experimental studies were carried out in parallel for a native fibril taken from rat’s tendon and heat-denatured fibril. Covering these two situations simultaneously allowed us to gain some insight on the relevance of the native quasi-crystalline structure of the fibril. We observed that in the temperature interval 59–75°C the native collagen fibril absorbs water (heating induced hydration) and stabilizes its Young’s modulus. These effects are clearly related to each other and are specific for the native fibril. According to the common-sense physical intuition, the water content was supposed to decrease under heating. We related these effects to activation of intermolecular interaction in the native fibril, and confirmed this hypothesis via studying the logarithmic decrement of damping. For temperatures lower than 59°C the water content is approximately constant, while the Young’s modulus decreases under heating. This effect is not specific to the native fibril and is related to reversible conformational changes that do not reorganize the layers of hydrated water in the fibril. For temperatures higher than 75°C, the Young’s modulus of the native sample increases with temperature. This is again in sharp contrast with the heat-denatured sample whose Young’s modulus steadily decreases with increasing the temperature. For these temperatures the water content and the logarithmic decrement of damping for the native fibril change non-monotonously. This intermediate state with a large Young’s modulus is meta-stable at sufficiently high temperatures: with time it slowly decays to the heat-denatured state thereby significantly decreasing its Young’s modulus.

On the theoretical side of the problem, we generalized the Zimm-Bragg model to account for the interaction between macromolecular chains confined in the fibril. The generalization proceeds via introducing the third state of a macromolecular unit (in addition to helical and coiled state already present in the standard Zimm-Bragg model). This intermediate state refers to inter-molecular structures. We have shown that besides the standard (single-molecule) Zimm-Bragg helix-coil transition, there is (at a lower temperature) a cooperative transition from the helical to the intermediate state. This transition is driven by inter-molecular interactions. During the transition one gets an additional influx of water to the first hydration layer. For higher temperatures the intermediate state gradually decays to the coiled state, while the water evaporates.

The strong correlation between the behavior of the Young’s modulus and the water content – the latter is stable when the Young’s modulus decreases, while the accumulation of water is accompanied by a constant Young’s modulus – may imply that mechanical features of collagen fibril regulate the amount of ambient water. This point does demand further experimental checks. If confirmed, it will present an example of a physical – in contrast to biochemical – mechanism of regulation. It is also tempting to suggest that the intermediate state plays a role in scar formation after burning. One clearly needs further research for (in) validating this suggestion.

## Supporting Information

Figure S1 **The schematic representation of the measuring chamber (seen from above).** 1: micro-tweezers (holder); 2: the studied sample; 3: thermostatted measuring chamber; 4: electrode which excites oscillations of the sample; 5: holder of the micro-tweezers; 6: lug of the air-mixer; 7: electromagnet; 8: magnetic conductor for exciting lug’s oscillations.
(TIF)

Figure S2 **The principal registration scheme of the sample oscillations.** 1: source of light; 2: mirror; 3: microscope capacitor; 4: micro-tweezers with the sample in the measuring chamber (not shown on the figure); 5: lens of the microscope; 6: field diaphragm; 7: photo-electronic multiplier; 8: oscillograph; 9: voltmeter of alternating voltage; 10: phasometer; 11: generator of alternating voltage; 12: source of constant voltage; 13: exciting electrode.
(TIF)

Text S1 **The measuring equipment described in some detail.**
(DOC)
