# Supplementary figures and images for: Stabilization and Anomalous Hydration of Collagen Fibril under Heating
Source: PLoS One. 2013 Nov 11;8(11):e78526. doi: 10.1371/journal.pone.0078526 (PMC3823754; doi:10.1371/journal.pone.0078526)

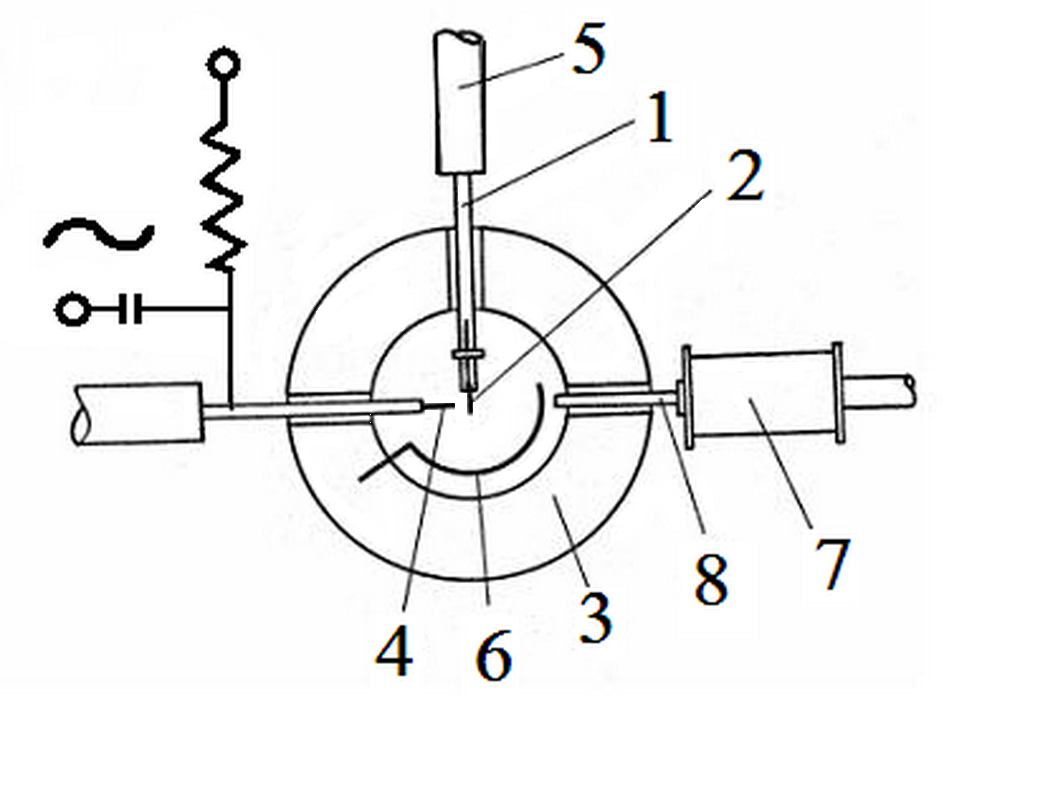

Supplement: Figure S1 — The schematic representation of the measuring chamber (seen from above). 1: micro-tweezers (holder); 2: the studied sample; 3: thermostatted measuring chamber; 4: electrode which excites oscillations of the sample; 5: holder of the micro-tweezers; 6: lug of the air-mixer; 7: electromagnet; 8: magnetic conductor for exciting lug’s oscillations. (TIF) [file pone.0078526.s001.tif]

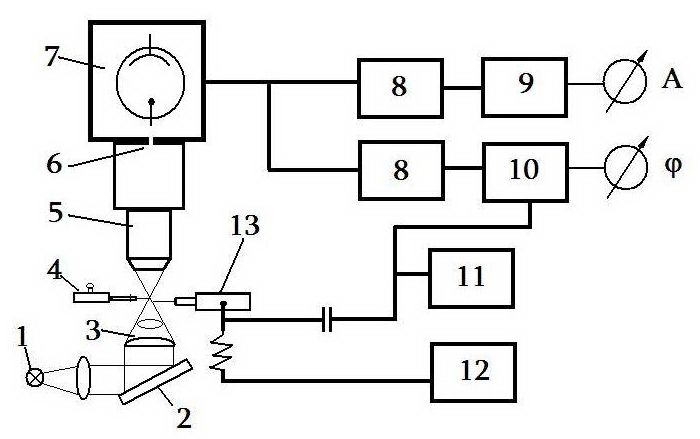

Supplement: Figure S2 — The principal registration scheme of the sample oscillations. 1: source of light; 2: mirror; 3: microscope capacitor; 4: micro-tweezers with the sample in the measuring chamber (not shown on the figure); 5: lens of the microscope; 6: field diaphragm; 7: photo-electronic multiplier; 8: oscillograph; 9: voltmeter of alternating voltage; 10: phasometer; 11: generator of alternating voltage; 12: source of constant voltage; 13: exciting electrode. (TIF) [file pone.0078526.s002.tif]
