# Supplementary material for: Stabilization and Anomalous Hydration of Collagen Fibril under Heating
Source: PLoS One. 2013 Nov 11;8(11):e78526. doi: 10.1371/journal.pone.0078526 (PMC3823754; doi:10.1371/journal.pone.0078526)
Supplement: Text S1 — The measuring equipment described in some detail. (DOC) [file pone.0078526.s003.doc]

***Supporting information for***

**Stabilization and anomalous hydration of collagen fibril under heating**

Sasun G. Gevorkian, Armen E. Allahverdyan, David S. Gevorgyan, Aleksandr L. Simonian, Chin-Kun Hu

**Legend:** In this Supporting Information we describe our measuring equipment is some detail**.**

The Young’s modulus and the logarithmic decrement of damping of collagen samples were measured in a thermostatted, humid chamber, which is schematically displayed on Fig. S1. Micro-tweezers (numbered as 1) with the sample (2) is placed into the measuring chamber on a special holder (5), which ensures the fixed orientation of the sample with respect to the the exciting electrode (4).

Oscillations of the sample (2) are induced by electrostatic forces from the electrode (4), which is subjected to a sinusoidal voltage. The electrode and the sample can be viewed as two facings of a capacitor, with their mutual attraction serving as the exciting force. For exciting the sample it suffices that it is polarized in the electrode field (or polarized randomly). To avoid a double-frequency exciting, the electrode was subjected to a constant shift voltage larger than the amplitude of the alternating voltage (~100 V).

Forced air mixing inside of the chamber was realized via lug (6) excited by electro-magnet (7) through magnetic conductor (8). The measuring chamber is fixed on the table of the microscope. To avoid temperature gradients and related dewfall for a large humidity, the microscope lens was also thermostatted.

The sample oscillations were registered photo-electrically; see Fig. S2. The beam of light from lamp (1), which was fed by a constant current, is reflected from mirror (2) and is focussed by the substage condenser (3) through the transparent bottom of the measuring chamber. The sample is fixed in tweezers (4). The microscope lens (5) (20X, digital aperture 0.40) projects the image of sample’s side surface on the field diaphragm (b), which is located in the back-focal plane of the lens. The diaphragm width is 80 mm. During the sample oscillations the shift of its image relative to the diaphragm changes the photocurrent of photo-electronic multiplier (7), which is located behind the diaphragm. The amplitude of the photo-current alternating component is registered via voltmeter (9) of the alternating voltage.

A part of the signal is transferred to the phasometer (10); see Fig. S2. The form of this signal is controlled by the oscillograph (8). Changing the frequency $\omega$ of the alternating voltage given to the exciting electrode, we find the resonance frequency $\omega_{0}$corresponding to the maximal signal amplitude. Normally, we observe only the main mode of oscillations, since other modes are too weak. Via the phasometer we register the frequency shift $\Delta\omega$, which in the resonance area corresponds to ±10°. These data can be employed for calculating the logarithmic damping decrement $\theta$.
